# Changes in Nematode Communities in Different Physiographic Sites of the Condor Seamount (North-East Atlantic Ocean) and Adjacent Sediments

**DOI:** 10.1371/journal.pone.0115601

**Published:** 2014-12-26

**Authors:** Daniela Zeppilli, Lucia Bongiorni, Ricardo Serrão Santos, Ann Vanreusel

**Affiliations:** 1 Centre of IMAR of the University of the Azores & LARSyS Associated Laboratory, PT-9901-862 Horta, Azores, Portugal; 2 IFREMER, Centre Brest, REM/EEP/LEP, Institut Carnot Ifremer-EDROME, ZI de la pointe du diable, CS10070, Plouzané, France; 3 Université de Brest, Institut Universitaire Européen de la Mer, Laboratoire des sciences de l'environnement marin (UMR6539 CNRS/IRD/UBO), Plouzané, France; 4 Institute of Marine Sciences – National Research Council (ISMAR-CNR), Arsenale - Tesa 104, Castello 2737/F, Venezia, Italy; 5 Department of Ocenography and Fisheries of the University of the Azores, PT-9901-862 Horta, Azores, Portugal; 6 Department of Biology, Marine Biology section, Ghent University, Krijgslaan 281, S8, Ghent, Belgium; Università di Genova, Italy

## Abstract

Several seamounts are known as ‘oases’ of high abundances and biomass and hotspots of biodiversity in contrast to the surrounding deep-sea environments. Recent studies have indicated that each single seamount can exhibit a high intricate habitat turnover. Information on alpha and beta diversity of single seamount is needed in order to fully understand seamounts contribution to regional and global biodiversity. However, while most of the seamount research has been focused on summits, studies considering the whole seamount structure are still rather poor. In the present study we analysed abundance, biomass and diversity of nematodes collected in distinct physiographic sites and surrounding sediments of the Condor Seamount (Azores, North-East Atlantic Ocean). Our study revealed higher nematode biomass in the seamount bases and values 10 times higher in the Condor sediments than in the far-field site. Although biodiversity indices did not showed significant differences comparing seamount sites and far-field sites, significant differences were observed in term of nematode composition. The Condor summit harboured a completely different nematode community when compared to the other seamount sites, with a high number of exclusive species and important differences in term of nematode trophic diversity. The oceanographic conditions observed around the Condor Seamount and the associated sediment mixing, together with the high quality of food resources available in seamount base could explain the observed patterns. Our results support the hypothesis that seamounts maintain high biodiversity through heightened beta diversity and showed that not only summits but also seamount bases can support rich benthic community in terms of standing stocks and diversity. Furthermore functional diversity of nematodes strongly depends on environmental conditions link to the local setting and seamount structure. This finding should be considered in future studies on seamounts, especially in view of the potential impacts due to current and future anthropogenic threats.

## Introduction

Seamounts are considered oases of the deep-sea life when compared with regular deep-sea environments [1]. It has been suggested that seamounts support high abundance and biomass and exhibit high species richness representing hotspots of diversity because high nutrient and food concentrations are available [2]. Such high production is driven by peculiar oceanographic conditions over seamounts, such as water turbulence and mixing, retention of nutrients and plankton and lateral advection of organic inputs [2]–[4]. The ‘oasis’ hypothesis originated from observations on higher abundance of filter feeders, such as corals and sponges, and commercially important fish in and over several seamounts [2], [3], [5]–[9]. In a study in the SW-Pacific Ocean, Rowden and colleagues [8] found four times higher epibenthic fauna biomass on seamounts than in adjacent slopes mainly due to the dominance of scleractinian corals. They concluded that besides their study provided some support for the seamount ‘oasis’ hypothesis, more investigations are needed targeting seamounts in less productive regions, with greater proportion of soft substrata and with less prevalent scleractinian corals population. Moreover, before the ‘oasis’ hypothesis can be extended to seamount invertebrates, more information on biomass for the macro- and meiofauna is required [1]. Recent studies on small invertebrates did not unequivocally support elevated standing stocks of the benthos on seamounts. For example, in the Condor Seamount (Azores, North-East Atlantic Ocean) highest values of meiofaunal abundance and biomass were found exclusively at the southern slope of the seamount, associated with specific oceanographic conditions [10]. In the Great Meteor Seamount Foraminifera occurred in very low densities compared with the surrounding area [11].

The “oasis hypothesis” supports also the idea of higher species richness in seamounts compared to the surrounding deep-sea ecosystems [2], [12]. Birds, mammals, turtles, fish and top pelagic predators are usually represented by high diversity over seamounts [13]. It is unclear, however, if increased habitat heterogeneity and complexity, for example due to the presence of biogenic structures, result in an elevated benthic diversity too [1]. The few studies available have shown little or no difference between seamount and non-seamount areas [8], [10], [11].

One of the priority issue related to the hypothesis of high seamount diversity is understanding pattern of beta diversity along the whole seamounts' structures. Beta diversity (defined as the variation in species composition among sites in a geographic area) is a key concept for understanding the functioning of ecosystems, for the conservation of biodiversity, and for ecosystem management [14]. The mechanisms of regional ecosystem stability can be understood by investigating the influence of ecological factors on alpha and beta variability [15]. Moreover, the analysis of the factors driving turnover diversity is crucial for a predictive understanding of the spatial patterns and species composition of deep-sea assemblages in different biogeographic regions [16]. Recent studies have shown that seamounts could exhibit a highly intricate turnover in habitats from their base to the summit [17], [18]. However so far most seamount research has focused on their summits and more rarely on upper slopes, while only few studies have been conducted on deeper flanks or bases [2], [19]–[21]. The low number of ecological studies considering the whole seamount structure therefore gives an incomplete picture of α and β diversity which is needed for any robust generalization about large-scale biodiversity patterns on seamounts [17], [21].

Seamount ecosystems are particularly sensitive and vulnerable to human disturbance and exploitation [19]. In the last years seamounts have been threatened by overfishing, trawling and mineral mining [19]–[22]. A fundamental part developing conservation strategies for threatened seamounts is to know the distribution, diversity and composition of all biological features likely to be impacted [22].

Meiofauna is, an important component of benthic communities, being characterized by high abundance, diversity and turnover rates [23]. Its relative contribution in abundance and biomass increases with increasing water depth, when compared with macrofauna [23], [24]. In the deep sea more than 90% of the total metazoan meiofaunal abundance is represented by nematodes [25]. Meiofauna and in particular nematodes are ideal model organisms for biodiversity studies as they are characterized by high species richness, recognizable feeding type, which offer the opportunity to examine patterns of structural and functional (trophic) diversity and different life-history strategies which have been shown to respond to environmental constraints [16], [26]–[28]. Moreover, the distribution of nematode along vertical sediment profiles can be related to differences in environmental conditions (i.e. sediment types, oxygen penetration; [29]). Despite an increasing knowledge on seamounts and their associated benthic biodiversity, our knowledge about small meiofaunal organisms is still rather poor [1], [10], [27], [30].

The main aim of the present study is to evaluate potential changes in nematode abundance, biomass, biodiversity levels, species composition and functional diversity in different physiographic sites of a single seamount (summit, flanks and bases). In this study we focused on a northeast Atlantic seamount, the Condor. In order to explore the potential differences between the seamount and the open-slope, sediments from the Condor were compared to those obtained in an external site (far field). We used the nematodes trophic traits (based on analysis of the feeding types classified according to the buccal morphology and size) and the maturity of nematode communities (based on life strategies) as proxies of nematodes functional diversity, assuming that these characteristics might affect nematodes functional roles ([2] and reference therein). In addition, we aimed at investigating how environmental constraints (including sediment grain size and available food resources) might affect nematodes across the whole seamount structure.

In particular we try to answer the following questions:

- *Are there differences in nematode community comparing distinct physiographic sites of the Condor Seamount*?
- *Is the Condor summit an area of higher nematode standing stock and a hotspot of diversity compared to other seamount habitats such as flanks and bases*?

## Materials and Methods

### Sampling and study area

**Figure 1:**
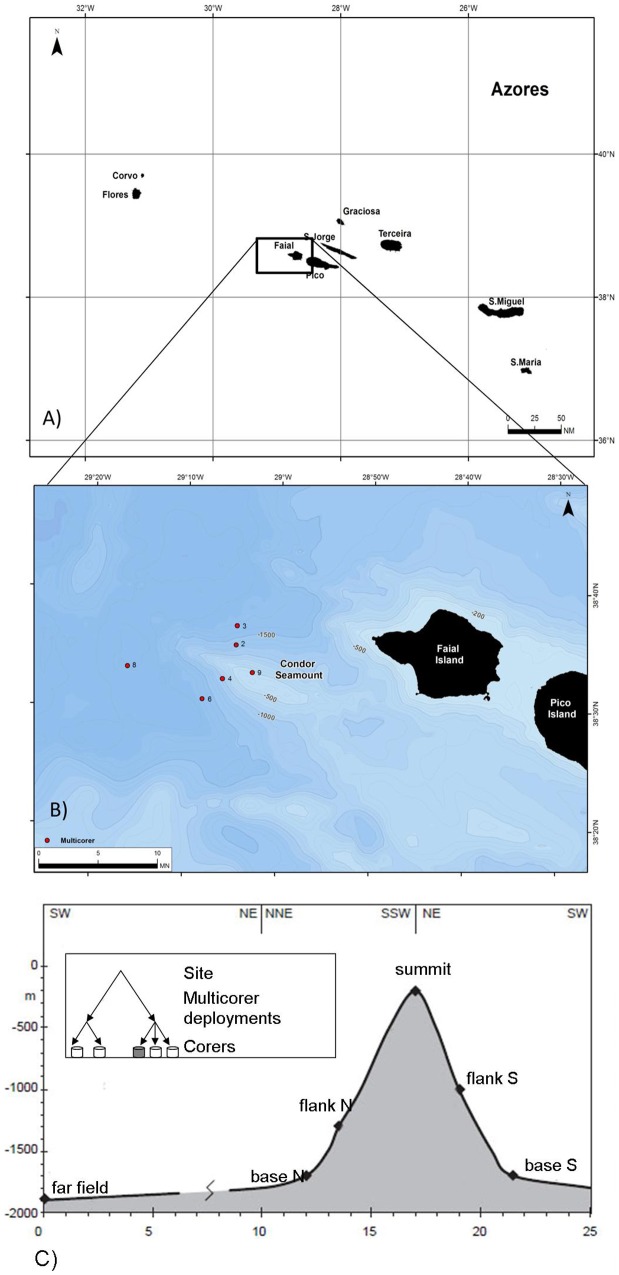
Map of the study area (A) and sampling sites (B): summit (site 9); flank North (site 2); base North (site 3); flank South (site 4); base South (site 6); far field (site 8). Schematic representation of the sampling design (C). Figure modified from [10].

The Condor Seamount is a linear volcano located in the archipelago of the Azores (northeast Atlantic), at ∼10 nm (nautical miles) southwest of the island of Faial (Fig. 1). This seamount presents a V-elongated shape and its depth ranges between 180 and 1700 m [31]. The summit displays large rocky seafloor outcrops, boulders and gravels, and the presence of coarse bioclastic deposits while the steep slopes are mainly characterized by unconsolidated sediments [31].

The seamount is mostly impacted by a dominant N-NW background oceanographic flow directed SE following a cyclonic rotation and hosts a multi-scale dynamic oceanographic conditions including enhanced mixing, upwelling-downwelling processes and closed circulation structures over the seamount, that make it distinct from the surrounding ocean [32]. The Condor Seamount hosts habitats of conservation importance, such as deep-water coral gardens and deep-sea sponge aggregations [31], [33].

Sediment samples for this study were collected in July 2010 during the Condor cruise of RV *Noruega*. A total of six sites were sampled. Five sites were located in correspondence with different physiographic features of the seamount: its summit (site 9, 206 m), northern flank (site 2, 1290 m), southern flank (site 4, 1006 m), northern base (site 3, 1687 m) and southern base (site 6, 1719 m). An additional site (chosen as external reference) was situated 10 nm SW the seamount (far-field, site 8, 1900 m) (Table 1 and Fig. 1). At all sites, sediments were collected with an interface multicorer (Midicorer Mark II 400) equipped with four core tubes (100 mm inner diameter), which allowed undisturbed and sealed sediment samples to be obtained [34]. The sampling strategy is described in details in Zeppilli et al. [10]. In each site the multicorer was deployed twice thus obtaining ten cores. Out of these four sediment cores (two from deployment 1 and two from deployment 2) were carefully subsampled for meiofaunal by inserting PVC liners (2.8 cm diameter). Two corers (one from each deployment) were used for analyses of organic matter and sediment characteristics. In order to describe sediment vertical distribution of nematodes, sediment cores were thin-sliced (0–1, 1–3, 3–5, 5–10, 10–15 cm), with exception of site 9 (summit) where the maximum corer penetration was 10 cm. All subsamples for meiofaunal analyses were placed in buffered 4% formalin solution and stained with Rose Bengal. This study did not involve endangered or protected species. This study did not involve vertebrates. This study work can be justified based on Portaria n° 48/2010 from 14 of May 2010, which basically establishes a scientific MPA aiming to enable a multidisciplinary integrated study of this seamount including habitats and biodiversity. This was done upon agreement of all stakeholders and interested parties. The area is public domain and under the legal administration of the Azores Government. The relevant regulatory was the Undersecretary of Fisheries of the Azores Regional Secretary of Environment and the Sea. No specific permissions were required for these locations/activities. Specific location of this study are: Site 9 (Summit) 38°32.94′N, 29°02.87′W; Site 2(Flank North) 38°35.26′N, 29°04.65′W; Site 4(Flank South) 38°32.28′N, 29°06.07′W, Site 3(Base North) 38°36.89′N, 29°04.59′W, Site 6 (Base South)38°30.65′N, 29°08.20′W; Site 8(Far-field) 38°33.30′N,29°16.30′W.

**Table 1:** Location and water depth of the sampling sites.

Sampling site	Description	Latitude (N)	Longitude (W)	Depth (m)
9	Summit	38° 32.94′	29° 02.87′	206
2	Flank North	38° 35.26′	29° 04.65′	1290
4	Flank South	38° 32.28′	29° 06.07′	1006
3	Base North	38° 36.89′	29° 04.59′	1687
6	Base South	38° 30.65′	29° 08.20′	1719
8	Far-field	38° 33.30′	29° 16.30′	1900

Methods in determining quantity and quality of organic matter in sediments, sediment characteristics and meiofaunal abundance, biomass and assemblages composition of the Condor Seamount and the far-field site corresponding to sampling sites of this study are detailed in Zeppilli et al. and Bongiorni et al. [10], [35].

### Nematode abundance and biomass

Briefly, sediment samples were pre-sieved through a 1000-µm-mesh net, and the organisms were retained on a 20-µm-mesh net. This latter fraction was resuspended and centrifuged three times with Ludox HS40 (density, 1.31 g cm^−3^ [34]). Nematodes were counted under a stereomicroscope and their biovolumes were measured only on intact specimens (371 nematodes in the summit, 331 nematodes in the flank North, 339 nematodes in the flank South, 316 in the base North, 310 in the base South, 342 in the far-field). The nematode biomass was calculated from the biovolume, which was estimated from all specimens per replicate using the Andrassy formula (V = L·W^2^·0.063·10^−5^, with body length, L, and width, W, expressed in µm [36]). The carbon contents were identified as 40% of the dry weight [37].

### Nematode biodiversity

From each sample, ca. 100 randomly selected nematodes were mounted on slides after formalin–ethanol–glycerol treatment to prevent dehydratation [34] and identified to the species level according to Platt & Warwick [38], [39], Warwick et al. [40], and the recent literature dealing with new nematode genera and species from the Atlantic Ocean. Unknown species were reported under the name of the Genus and then as sp1, sp2 and so on.

Nematode species richness (NSR) was calculated as the total number of species collected for each site. Nematode species diversity (H′, using log-base e) was measured using the Shannon–Wiener diversity index, with the evenness as Pielou Index (J). The Margalef diversity index (D) was estimated as D = (S-1/ln N), where S is the number of nematode species and N is the number of individuals in the sample. In order to facilitate the comparison among samples the expected number of nematode species for a theoretical random sample of 100 individuals, ES (100), was calculated. All indices were calculated using PRIMER6 software (Plymouth Marine Laboratory, UK [41]).

We also measured the turnover among samples (β diversity). The β diversity provides indications of any change in species composition among the samples [16] and can be expressed as percentages of dissimilarity of nematode community species composition (e.g. calculated on a Bray–Curtis similarity matrix [42]). The SIMPER analysis was used to determine the contributions of each species to the average Bray-Curtis dissimilarity [43]. Before the analysis, the diversity matrix was square root transformed. The trophic diversity of the nematodes was determined by analysis of the trophic groups, as reported by Wieser [44]. The nematodes were divided into four original groups, as follows: (i) no buccal cavity or a fine tubular one, as selective (bacterial) feeders (1A); (ii) large but unarmed buccal cavity, as non-selective deposit feeders (1B); (iii) buccal cavity with scraping tooth or teeth epistrate or epigrowth, as diatom feeders (2A); and (iv) buccal cavity with large jaws, as predators/omnivores (2B). The Index of Trophic Diversity (ITD) was calculated as θ, where θ = g_1_^2^+g_2_^2^+g_3_^2^…+g_n_^2^, and g is the relative contribution (in terms of number of specimens) of each trophic group to the total number of individuals, and n is the number of trophic groups ([45] and literature therein). For n = 4, θ ranges from 0.25 (highest trophic diversity; i.e. the four trophic groups account for 25% of the nematode abundance each) to 1.0 (lowest diversity; i.e. when one trophic group accounts for 100% of the nematode abundance). Nematode trophic structure was calculated on nematode biomass matrix.

The nematode life strategies (r - k) were described by the maturity index (MI) of the nematodes for which life strategies are known. In order to identify colonization strategies, nematodes are divided into “colonizers” (comparable to r-strategists, characterized by short life cycle, high colonization ability, and tolerance to disturbance, e.g. eutrophication, and anoxybiosis) and “persisters” (k-strategists with low reproduction rate, long life cycle, and low colonization ability and tolerance to disturbance; the list of species with different life strategies is reported by Bongers et al. [46]). The MI was calculated according to the weighted mean of the individual genus scores: MI = Σ v (i) X f (i), where v is the c–p value (colonisers– persisters; ranging from 1, i.e., only opportunistic colonizers to 5, i.e., only persisters) of the genus i [46] and f (i) is the frequency of that genus.

### Statistical analyses

Differences in nematode abundance, biomass and biodiversity among sites (including seamount and in the external area) were tested by a one-way analysis of variance (ANOVA). The GMAV (1997) statistical package (University of Sydney, Australia) was used to perform the ANOVA. Before the ANOVA, the homogeneity of variances was tested using the Cochran test and data were appropriately transformed whenever necessary. For those data which transformation did not allow a homogenization of variance, we adopted a more conservative level of significance. The Student Newman-Keuls (SNK) test was used for post hoc comparisons.

Bray-Curtis similarities among all of the sampling sites (with data fourth root transformed), the analysis of the similarities (ANOSIM) and the similarity percentages (SIMPER) were performed with the PRIMER6 [41]. These statistical analyses were carried out to measure the similarities in the nematode specie composition among all of the investigated samples (24 replicates: 6 sites X 4 replicates).

To evaluate the relationship between nematode abundance, biomass and species composition and environmental variables (i.e. water depth, sediment grain size as indicator of habitat heterogeneity, and trophic characteristics of sedimentary organic matter as proxy of food availability for benthic consumers) we conducted a non-parametric multivariate multiple regression analysis (DistLM: distance-based linear model) using the PERMANOVA + add-on package for PRIMER6 software [47], [48]. This method analyses and models the relationship between a multivariate data cloud, and one or more predictor variables. It is based on a resemblance matrix and uses permutations, rather than the restrictive Euclidean distance and normality assumptions which underlie the standard approach to linear modelling. For total abundances, biomasses diversity indices the Euclidean distance was used as resemblance measure, whereas for species composition the analysis was based on Bray-Curtis dissimilarities. The forward selection was carried out and the adjusted R^2^ was selected as criterion to enable the fitting of the best explanatory environmental variables in the model [48]. The results are provided as marginal and sequential test. The marginal test revealed how much each variable explains when taken alone, ignoring all other variables. Following the results of this test a sequential test was performed which examines whether the addition of that particular variable contributes significantly to the explained variation [48]. Only variables (nematode variables, sediment parameters and trophic resources) related to the 0–1 cm sediment layers were tested. Quantity and quality of organic matter in sediments, sediment grain size characteristics of the Condor Seamount and the far-field site corresponding to sampling sites of this study are detailed in Zeppilli et al. and Bongiorni et al. [10], [35]. Concentrations of phytopigments and biopolymeric organic carbon (BPC, as sum of protein, carbohydrate and lipid carbon equivalents [49]) were used as indicators of the amount of trophic resources while the protein to carbohydrate concentrations ratio (PRT:CHO) was used as indicators of their quality. Percentage of gravel, sand, silt and clay in the sediment were used as indicator of habitat heterogeneity [50]. For the grain size variables, the same values of each parameters was assigned to all four faunal cores from the same site, while in the case of the trophic variables, four independent values of each parameter were available. Water depth was used as additional environmental constraint.

## Results

### Nematode abundance and biomass

Nematodes dominated meiofaunal abundance at all sampling sites (85–93%, for details see Zeppilli et al. [10]). Nematode abundance was significantly higher at the southern flank than at the far-field site (340.6±150.7 and 159.3±34.2 individuals per 10 cm^2^, respectively, Table 2 and 3; p<0.05), while nematode biomass at northern and southern bases was significantly higher than at the other seamount sites and 10 times higher than at the far-field (p<0.005; Tables 2 and 3). Nematode abundance and biomass were generally concentrated in the top first centimeter-layer of the sediment cores and decreased with depth along the vertical profiles, except for the summit, where distribution was homogeneous among layers (for details see Zeppilli et al. [10]).

**Table 2:** Nematode abundance, biomass and indices of diversity at all sampling sites in the Condor Seamount and in the far-field site.

	Abundance (ind 10 cm^−2^)		Biomass (µgC10 cm^−2^)		NSR		D		J		ES (100)		H′		ITD		MI	
	avg	Sd	avg	sd	avg	sd	avg	sd	avg	sd	avg	sd	avg	sd	avg	sd	avg	sd
Summit	218.30	45.25	13.96	8.26	34.75	4.86	7.06	1.06	0.81	0.12	31.73	4.88	2.88	0.47	0.59	0.14	3.59	0.22
Flank North	183.51	47.79	7.30	2.16	31.00	10.52	6.85	1.95	0.88	0.04	30.85	10.35	3.00	0.38	0.58	0.12	3.43	0.04
Flank South	340.55	150.65	19.07	12.14	40.50	7.42	8.11	1.46	0.85	0.04	35.24	5.58	3.13	0.26	0.59	0.06	3.55	0.09
Base North	254.07	75.79	89.69	33.33	43.00	5.35	8.74	0.98	0.90	0.02	39.24	3.30	3.37	0.16	0.39	0.05	3.23	0.12
Base South	288.86	74.58	43.62	14.28	43.00	7.87	8.90	1.37	0.87	0.06	39.45	6.14	3.25	0.35	0.37	0.03	2.90	0.15
Far-Field	159.25	34.23	6.86	2.05	37.50	2.08	8.29	0.13	0.93	0.02	37.46	2.01	3.36	0.06	0.41	0.07	3.19	0.09

Reported are Nematode Species Richness (NSR), the index of Margalef (D), Pielou (J), the expected species number ES(100), Shannon–Wiener (H′), the index of trophic diversity (ITD) and the maturity index (MI). Avg  =  average, sd  =  standard deviation.

**Table 3:** Output of the one-way ANOVA carried out to test for differences of all of the variables investigated among all seamount and far-field sites.

Variables	d.f.	MS	F	P		Output of the SNK test
Nematode abundance	5	17.6755	3.16	0.0322	*	Flank S > Far-field
Nematode biomass	5	26.7486	21.56	0.0000	***	Base N > Base S > Others
SR	5	92.4417	1.95	0.1354	n.s.	-
D	5	2.9266	1.77	0.1694	n.s.	-
J	5	0.0063	1.81	0.1609	n.s.	-
ES(100)	5	55.3736	1.55	0.2248	n.s.	-
H′	5	0.1555	1.60	0.2117	n.s.	-
ITD	5	0.0477	6.31	0.0015	**	Summit, Flank N and Flank S > others
MI	5	0.2730	16	0.0000	***	Summit, Flank N and Flank S > Base N and Far-field > Base S

d.f., degree of freedom; MS, mean square; F, F ANOVA statistic; P, probability level: ***P<0.001; **P<0.01; *P<0.05; n.s., not significant.

### Nematode diversity

The nematode species richness, Shannon–Wiener, Margalef and Pielou, trophic diversity and maturity indexes as well as the expected number of nematodes species for all sites investigated are reported in Table 2. Overall, 251 nematode species belonging to 116 genera and 25 families were identified. In all sites, Desmoscolecidae was the most abundant family, accounting for 28.4–63.0% of the total nematode abundance, except for the summit, where the Epsilonematidae was the dominant family, accounting for 38.7% of the total nematode abundance (Table 4). Only 9 families occurred in all sites. The family Epsilonnematidae, Encellidae, Siphonolaimidae, Aponchiidae, and Draconematidae were exclusively found on the seamount while no exclusive families were encountered in the far-field site (Table 4). The family Aponchiidae were found only in the northern flank, while Siphonolaimidae was exclusive present at the southern base (Table 4).

**Table 4:** Nematode families (% on total abundance) at the investigated sampling sites.

Base N		Flank N		Summit		Flank S		Base S		Far-Field	
Desmoscolecidae	43.1	Desmoscolecidae	48.5	Epsilonematidae	38.7	Desmoscolecidae	63.0	Desmoscolecidae	28.4	Desmoscolecidae	36.4
Comesomatidae	17.5	Leptolaimidae	8.6	Selachinematidae	22.5	Sphaerolaimoidea	7.7	Comesomatidae	27.5	Sphaerolaimoidea	15.6
Chromadoridae	9.9	Chromadoridae	7.6	Desmoscolecidae	13.5	Desmodoridae	4.7	Desmodoridae	8.2	Chromadoridae	9.9
Sphaerolaimoidea	6.5	Oxystominidae	6.3	Draconematidae	8.3	Oxystominidae	4.5	Sphaerolaimoidea	8.2	Oxystominidae	7.0
Desmodoridae	4.7	Desmodoridae	5.0	Desmodoridae	4.4	Chromadoridae	4.3	Chromadoridae	5.8	Ceramonematidae	6.6
Oxystominidae	4.5	Selachinematidae	3.3	Chromadoridae	2.5	Leptolaimidae	3.4	Oxystominidae	5.4	Comesomatidae	4.6
Leptolaimidae	3.9	Comesomatidae	2.7	Ceramonematidae	2.1	Comesomatidae	2.2	Leptolaimidae	4.2	Leptolaimidae	4.3
Ironidae	3.0	Phanodermatidae	2.7	Oxystominidae	2.1	Camacolaimidae	1.4	Microlaimidae	2.3	Linhomoeidae	2.6
Selachinematidae	2.4	Sphaerolaimoidea	2.7	Cyatholaimidae	1.9	Ironidae	1.4	Selachinematidae	2.1	Desmodoridae	2.3
Ceramonematidae	1.9	Cyatholaimidae	2.7	Microlaimidae	1.2	Selachinematidae	1.4	Ceramonematidae	1.9	Camacolaimidae	2.0
Cyatholaimidae	0.6	Microlaimidae	2.0	Sphaerolaimoidea	1.2	Ceramonematidae	1.2	Camacolaimidae	1.2	Ironidae	2.0
Phanodermatidae	0.6	Diplopeltidae	1.7	Comesomatidae	0.4	Microlaimidae	1.0	Cyatholaimidae	1.2	Monhysteridae	2.0
Camacolaimidae	0.4	Ceramonematidae	1.3	Oncholaimidae	0.4	Diplopeltidae	0.8	Phanodermatidae	0.9	Diplopeltidae	1.4
Diplopeltidae	0.3	Epsilonematidae	1.3	Thoracostomopsidae	0.4	Cyatholaimidae	0.6	Diplopeltidae	0.7	Selachinematidae	1.4
Epsilonematidae	0.2	Aponchiidae	0.7	Enchelidiidae	0.2	Enchelidiidae	0.6	Ironidae	0.5	Thoracostomopsidae	0.7
Linhomoeidae	0.2	Enchelidiidae	0.7	Monhysteridae	0.2	Monhysteridae	0.6	Monhysteridae	0.5	Cyatholaimidae	0.3
Oncholaimidae	0.2	Ironidae	0.7			Epsilonematidae	0.4	Enchelidiidae	0.2	Microlaimidae	0.3
		Linhomoeidae	0.7			Phanodermatidae	0.4	Epsilonematidae	0.2	Oncholaimidae	0.3
		Camacolaimidae	0.3			Draconematidae	0.2	Linhomoeidae	0.2	Phanodermatidae	0.3
		Draconematidae	0.3			Linhomoeidae	0.2	Siphonolaimidae	0.2		
		Monhysteridae	0.2					Thoracostomopsidae	0.2		

**Figure 2:**
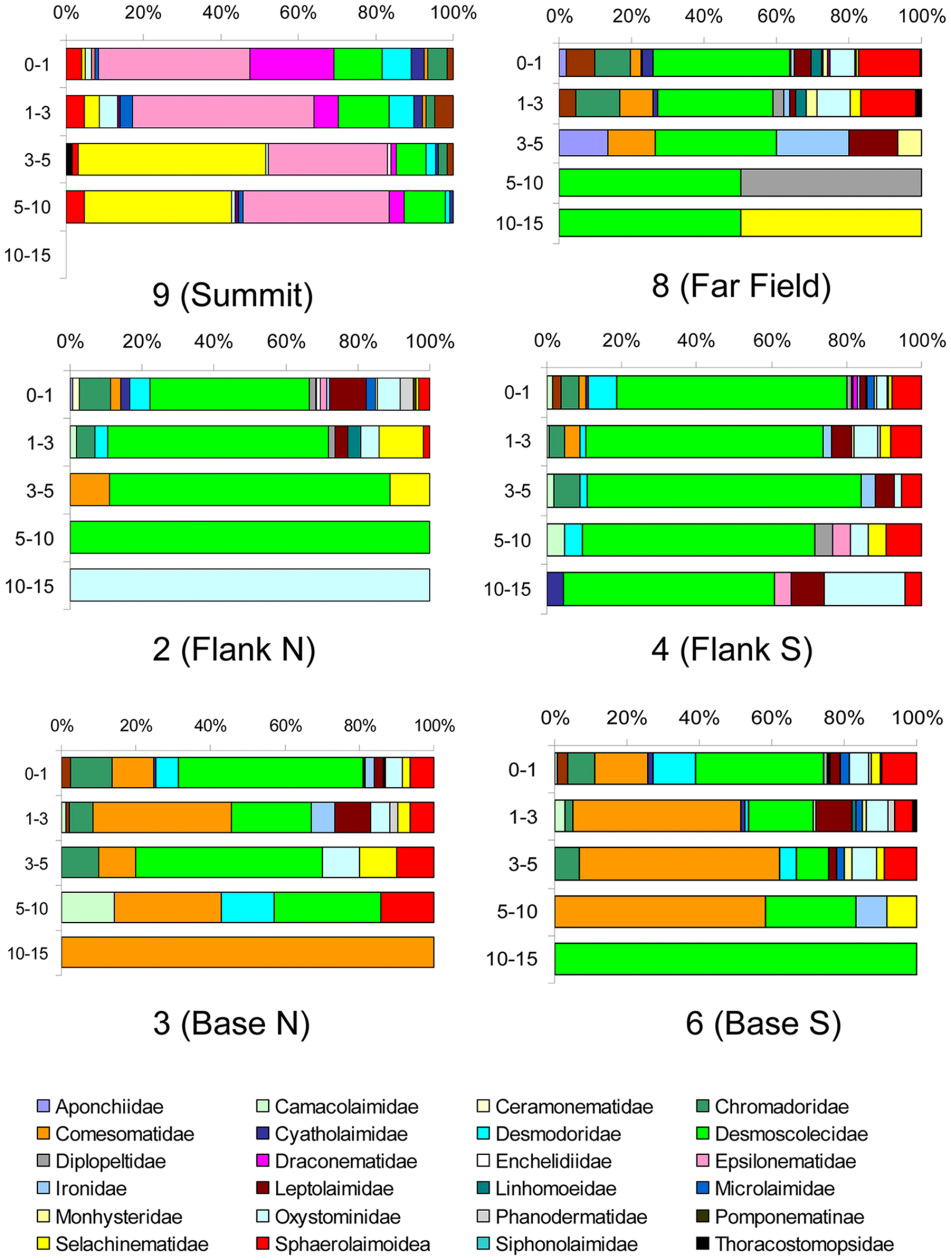
Vertical profiles of the nematode family in the sediments. n.a. not available.

In the summit, the family Epsilonematidae dominated the sediments from the surface up to the first 3 cm depth, while Selachinematidae dominated the sediment layers from 3 to 10 cm depth (Fig. 2). In both seamount flanks, Desmoscolecidae dominated the 0–10 cm layers representing from 56.5 to 73.2% of the abundance, while only the Oxystominidae were present from 10 to 15 cm sediment depth. At the base North, Desmoscolecidae dominated the 0–1 cm and 3–5 cm sediments layers, while Comesomatidae dominated the 1–3 and the 5–15 cm layers. In the base South, Desmoscolecidae dominated the 0–1 and 10–15 cm layers, while Comesomatidae dominated the other sediment layers. Desmoscolecidae dominated all the sediment layers in the far-field, representing from 31.8 to 50% of the total nematode abundance.

In the Condor Seamount, the nematode species richness ranged from 31.0±10.5 to 43.0±7.9 (in the flank North and the base South, respectively), while in the far-field site nematode species richness was 37.5±2.1. However no significant differences, in term of NSR and diversity indices, where encountered when comparing different seamount areas and the outer far-field site (Tables 2 and 3).

Out of the 251 nematode species encountered in the investigated areas, 160 (63.7%) were exclusive of the seamount, whereas 25 (10.0%) species were encountered only in the external far-field site. Comparing different sites within the seamount 35 species were encountered exclusively in the summit (representing 13.9% of the total nematode species richness), 12 species were encountered only in the flank North (4.8%), 13 species were encountered only in the flank South (5.2%), 12 species were encountered only in the base North (4.8%) and 19 species were exclusively encountered in the base South (7.6%).

**Figure 3:**
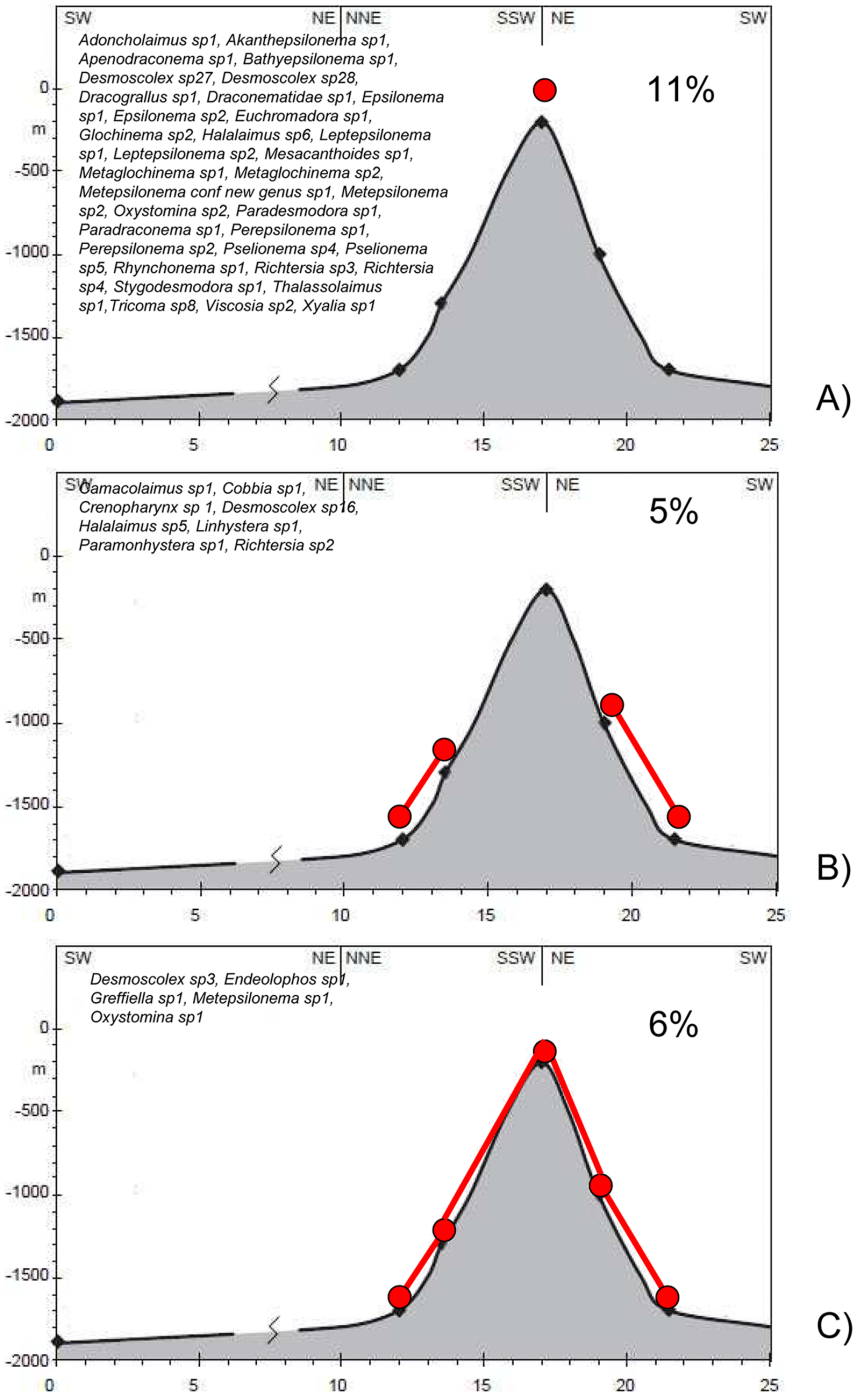
Distribution of nematode species in the Condor Seamount. Species restricted to the summit (A), species restricted to the flanks and the bases (B) and species distributed in all the seamount (C). Reported are the list of the species and the relative percentage of each species group to the total nematode abundance.

Several species showed a clear bathymetric distribution along the seamount structure: 35 species, representing 11% of the Condor Seamount nematode abundance were restricted to the summit (Fig. 3a), while 8 species (5% of the Condor Seamount nematode abundance) were restricted to the flanks and the bases (Fig. 3b). Only 5 species, representing 6% of the Condor Seamount nematode abundance, were widely distributed in all the Condor sites (Fig. 3c).

**Figure 4:**
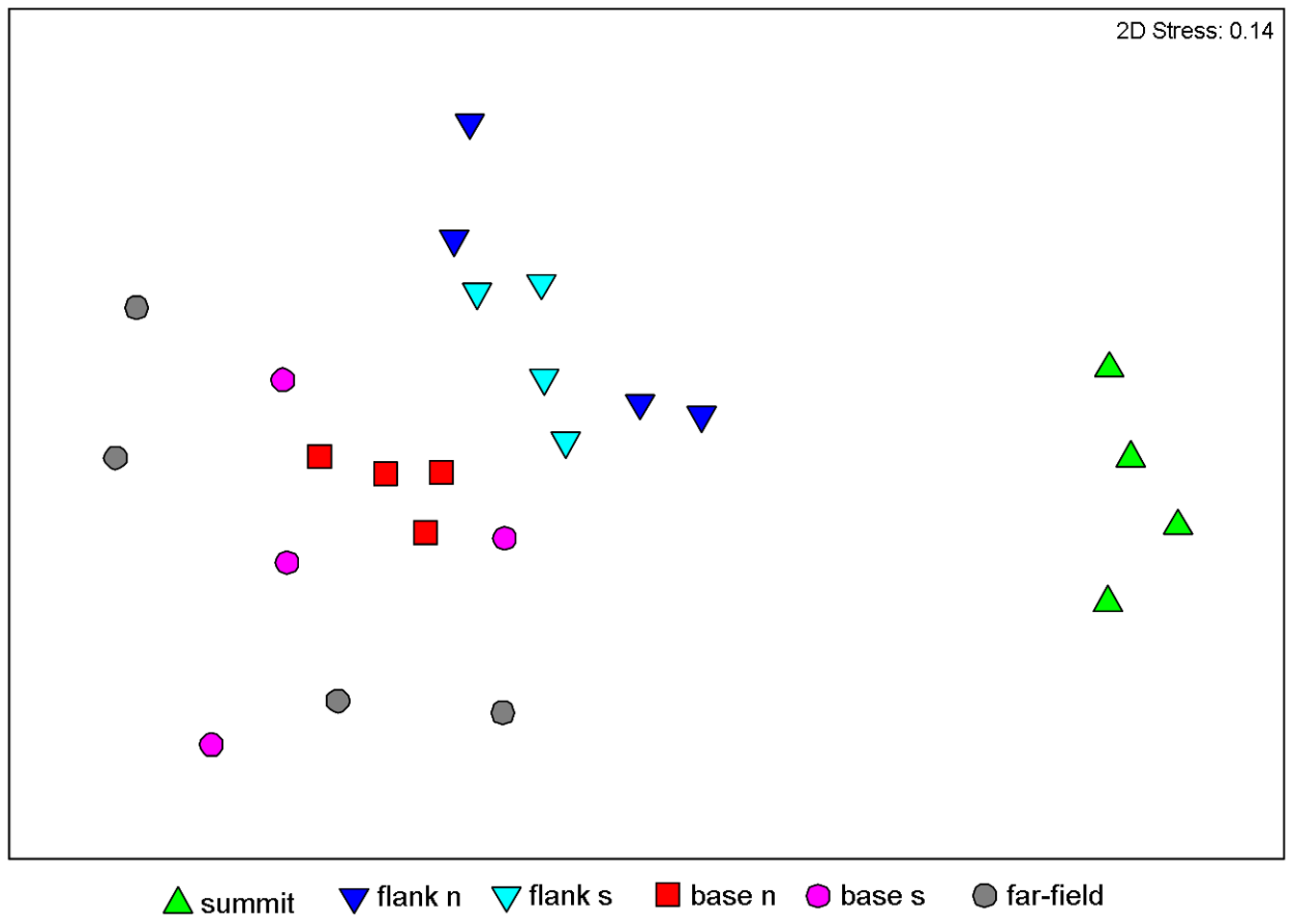
Multi-dimensional scaling analysis performed using species composition.

**Figure 5:**
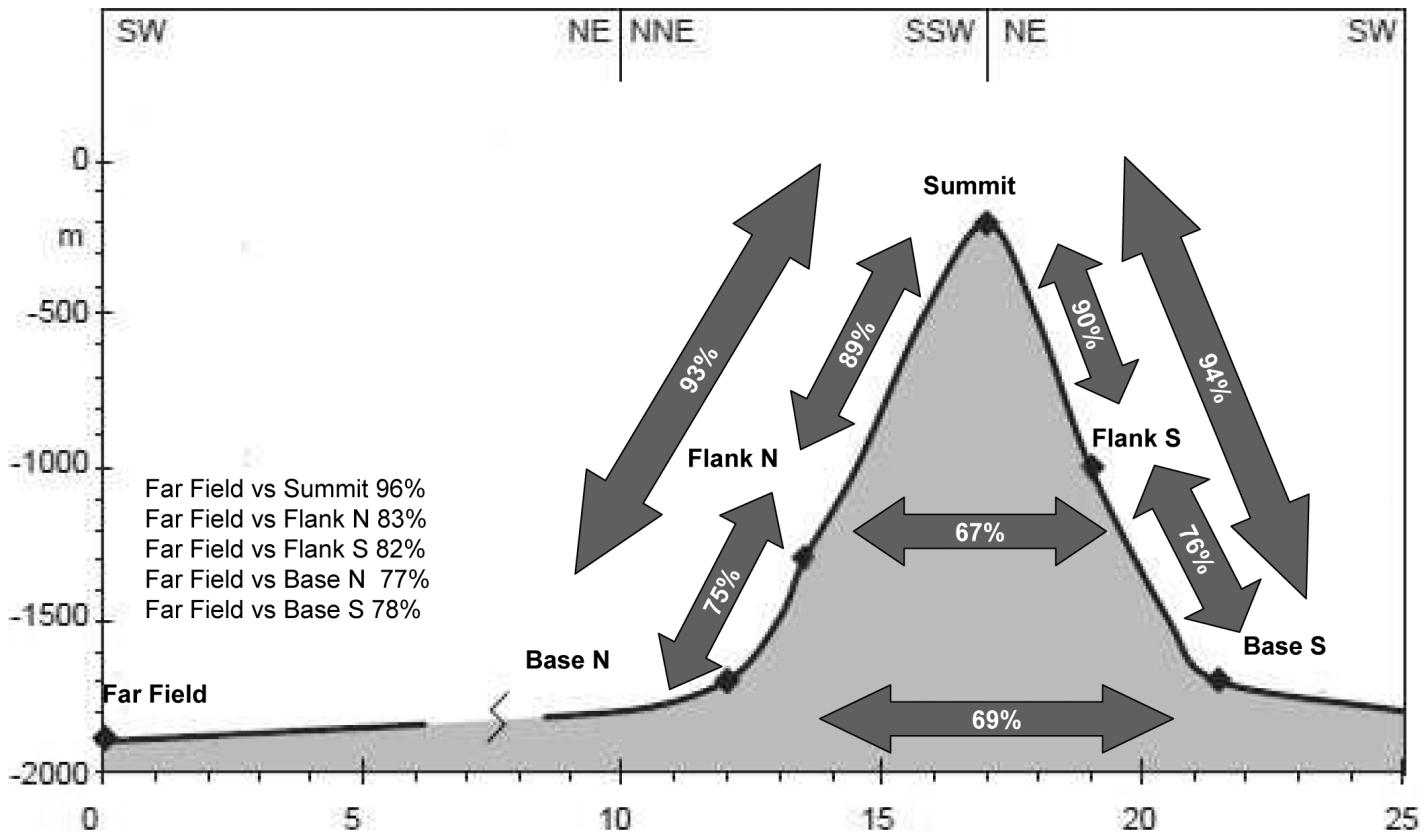
Nematode beta-diversity in the different investigated sites.

**Figure 6:**
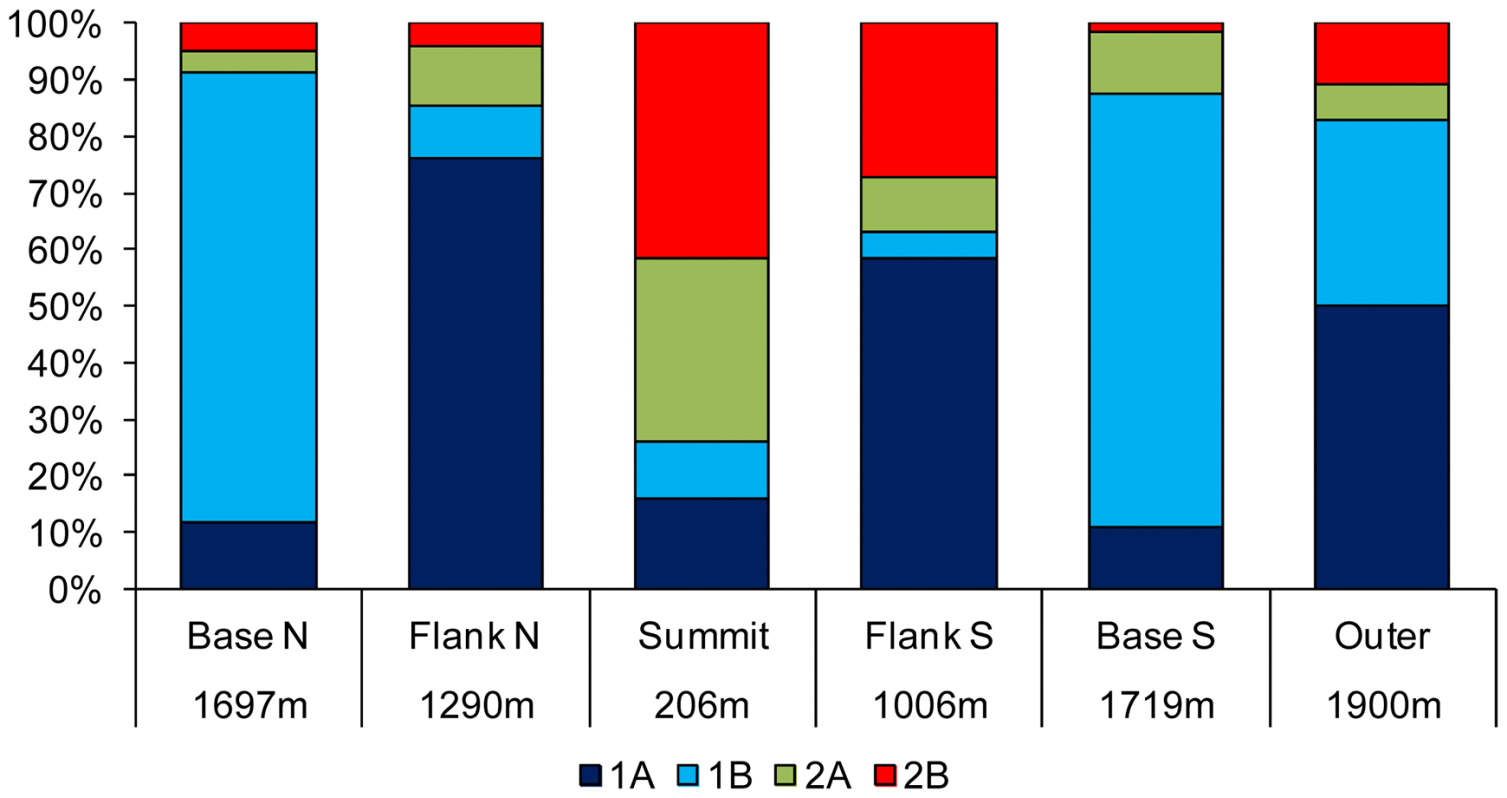
Nematode trophic structure calculated on nematode biomass values. 1A: selective (bacterial) feeders; 1B: non-selective deposit feeders; 2A: epistrate feeders; 2B: predators/omnivores.

The multidimensional scaling analysis combined with % similarity cluster lines based on nematode species composition revealed that samples from the summit were clearly separated from the other sites (Fig. 4). A high dissimilarity characterized replicates of the far field. Interestingly the analyses revealed that some replicates of the far field site resembled those of the southern base rather than themselves. The SIMPER and ANOSIM analyses revealed significant differences in the nematode species composition among all sites (Table 5). Nematodes turnover (β-diversity) among seamount samples was high ranging between 67 and 94%. The higher values were observed between the summit and the bases. The highest β-diversity was observed between species inhabiting the seamount summit and those in the far-field site (96%, Fig. 5). In all areas, with the exception of the summit, deposit feeders (including selective and non-selective feeders) ranged from 63.3% to 91.3% and dominated the trophic structure of the nematode biomass (Fig. 6). However, while on seamount flanks selective deposit feeders were more abundant, in both seamount bases the non-selective ones prevail. In the seamount summit predators, mainly represented by two species *Adoncholaimus sp.* and *Mesacanthoides sp.*, were dominant (41.3%), while epistrate feeders represented a conspicuous percentage (32.7%). The index of trophic diversity ranged from 0.37±0.03 to 0.59±0.14 and was significantly higher in the summit and flanks compared to the bases and the far-field site (Table 2 and Table 3). The Maturity Index ranged between 3.6±0.2 in the summit and 2.9±0.2 in the base South (Table 2 and 3). The MI was significantly higher in the summit and flanks than in the bases and in the far-field site (p<0.001; Table 3).

**Table 5:** Results of the ANOSIM and SIMPER analyses for differences in the nematode community structures.

	ANOSIM		SIMPER
	*R*	*p*	Dissimilarity %
Summit vs Flank N	0.979	0.029	89
Summit vs Flank S	1	0.029	90
Summit vs Base N	1	0.029	93
Summit vs Base S	1	0.029	94
Summit vs Far-Field	1	0.029	96
Flank N vs Flank S	0.427	0.029	67
Flank N vs Base N	0.823	0.029	75
Flank N vs Base S	0.771	0.029	81
Flank N vs Far-Field	0.823	0.029	83
Flank S vs Base N	0.896	0.029	67
Flank S vs Base S	0.708	0.029	76
Flank S vs Far-Field	0.948	0.029	82
Base N vs Base S	0.354	0.029	69
Base N vs Far-Field	0.719	0.029	77
Base S vs Far-Field	0.563	0.029	78

### Multiple correlations with environmental variables

The DistLM analysis allowed the identification of the environmental variables that were best correlated to the observed nematode distribution patterns (Table 6). The differences in nematode abundance were significantly explained for 25.5% by water depth, while the differences in nematode biomass were manly explained by protein to carbohydrate ratios (45.1%), followed by phytopigments (26.9%) and only for a small fraction by water depth (8.2%). The differences in the nematode species composition were significantly explained by sediment characteristics (water content and percentage of gravel and sand; total of 42.5%) and by phytopigments (5%). Sediment characteristics (percentage of sand) significantly explained 57.2% of the differences in the Index of Trophic Diversity, while phytopigments (55.3%), percentage of sand and gravel (19.0%) and water depth (7.3%) explained the Maturity Index differences.

**Table 6:** Results of the DistLM analysis.

	Variables	SS	F	P		Variance (%)
Nematode abundance	Water depth	53590	8.0561	0.006	**	25.5
	Phytopigments	16736	1.9049	0.213	n.s.	8.0
	Sediment water content	15975	2.5825	0.113	n.s.	7.6
	Percentage of sand	3409.1	0.5384	0.463	n.s.	1.6
	PRT:CHO	1788.5795	0.2716	0.635	n.s.	0.9
						
Nematode biomass	PRT:CHO	11420	18.0732	0.002	**	45.1
	Phytopigments	6817.229	20.209	0.001	**	26.9
	Water depth	2076.401	8.2927	0.014	*	8.2
	Percentage of silt	390.169	1.6054	0.209	n.s.	1.5
	Percentage of sand	0.8895	0.0035	0.95	n.s.	0.0
						
Nematode species composition	Sediment water content	15413	6.549	0.001	**	22.9
	Percentage of gravel	7607.6	3.617	0.001	**	11
	Percentage of sand	5780	3.0113	0.001	**	8.6
	Phytopigments	3359.8	1.8224	0.007	**	5
	Water depth	2484.1	1.3739	0.098	n.s.	3.7
						
Index of Trophic Diversity	Percentage of sand	0.2143	29.359	0.001	**	57.2
	Phytopigments	0.0059	0.808	0.346	n.s.	1.6
	Percentage of gravel	0.0036	0.4797	0.462	n.s.	1.0
	Water depth	0.0119	1.6206	0.209	n.s.	3.2
	Percentage of clay	0.003	0.3922	0.568	n.s.	0.8
						
Maturity Index	Phytopigments	0.9253	27.2585	0.001	**	55.3
	Percentage of sand	0.1706	10.543	0.007	**	10.2
	Percentage of gravel	0.1466	5.129	0.022	*	8.8
	Water depth	0.1221	5.107	0.041	*	7.3

Selection criterion: adjusted R^2^. highlighting the effect of different variables on nematode abundance, biomass and biodiversity (SS, sum of squares; F, F statistic; P, probability level;

***P<0.001;

**P<0.01;

*P<0.05, n.s., not significant).

## Discussion

Seamounts are considered oases of marine faunal standing stock and biodiversity if compared to surrounding deep-sea habitats [1]. Considering seamounts ecology, these structures have been described as a mosaic of habitats harboring astounding densities of filter feeders [5], fishes [1] and high level of benthic biodiversity [2]. However a critical point to the hypothesis of heightened seamounts biodiversity relies on the lack of knowledge on density and diversity patterns, and in particular on information on alpha and beta diversity across individual seamounts structures (from their summit along flanks to bases). Only a deeper understanding of those patterns might help to elucidate large scale biodiversity gradient on seamounts [17] and their contribution to regional and global biodiversity.

In line with the general knowledge that nematode abundance and biomass decrease with increasing bathymetric depth [51], we observed a relatively low nematode standing stock on the Condor Seamount summit, while the highest abundance and biomass were observed on its deepest sites (the southern flank, and both N and S bases). Our findings are in contrast with what has been observed for macrofauna on two northeast Atlantic seamounts (Senghor and Condor [18], [35]) suggesting that macrofauna and meiofauna may respond differently to local environmental constraints. Several studies showed that the local environment may affect nematode total biomass, both in coastal and in deep-sea environments [52], [53] and food availability is considered as one key environmental factor shaping density patterns of meiobenthic fauna [54]. Data from this study are mainly in accordance with levels of food resources for benthic consumers as the organic matter concentration in the Condor sediments was observed to increase from the summit toward its bases [35]. Indeed the trophic conditions (expressed as quality of the sediment organic matter) explained a significant portion of variation in the Condor nematode biomass (45.1%, Table 6). Processes such as sweeping off of seamounts' summits by strong currents and the presence of shallow cyclonic circulation patterns have been hypothesized to be responsible for the frequently observed depletion of organic matter in shallower sites of the Condor and other mounts [35], [55]. Although this study is limited to one external area, when comparing the Condor Seamount with the off-mount sediments, we found higher nematode biomass at the seamount bases than in far-field site at comparable depth. The nematode biomass values recorded in the Condor bases were consistent with other enriched deep-sea habitats such as the Gollum Channels and the Whittard Canyon (NE Atlantic [56]). However, our data are in contrast with a previous investigation on two Mediterranean seamounts [57] where meiofaunal biomass (mainly composed by nematodes) was generally lower in the sediment close to the seamounts than in surrounding deep-sea sediments away from the mounts. This difference can be related to the level of food resources (biopolymeric C and phytopigments) that are very low at the basis of the Mediterranean seamounts, and high at the basis of the Condor Seamount (see Bongiorni et al. [35] for details). Besides the fact that the amount of food supplies to organisms could explain differences between communities in mounds and slopes, it is likely that taxa responsible for such a difference will be those that are more able to use local resources. In their study, Rowden and colleagues [8] reported higher levels of megafauna biomass on twenty SW Pacific seamounts compared with adjacent slopes. These differences were mainly due to the dominance of a filter feeder coral species, known to be efficient in exploiting particulate organic matter. In our study the 10 times higher nematode biomass found at the Condor bases (Table 2) was mainly explained by the presence of the non-selective deposit feeders species *Comesomoides sp.*, particularly abundant (13% of the nematode abundance) in both seamount sites. The average biomass for individuals of this species (0.59 µgC) was 10 times higher than the average biomass reported for the other Condor nematodes (0.05 µgC).

Nematode diversity values encountered in the Condor are comparable to data available on this region at similar water depth [16]. Surprisingly we did not find difference in number of nematode species, ES(100) or other biodiversity indices among seamount sites and with those of the adjacent continental slope. Despite the lack of clear bathymetric or N-S diversity pattern across the seamount, several nematode species on the Condor showed a specific bathymetric distribution (Fig. 3). In addition, we found high differences in the species compositions among seamount sites reflecting clear cut differences along bathymetric gradient and flanks orientation (dissimilarity range between 67 and 94%, Fig. 5). These differences result from a large fraction of the nematode species being exclusively associated with the sediments of different seamount sites. In particular the summit harboured a peculiar nematode community (Fig. 4), where ca 14% of the species was exclusive of this site. On the Condor summit we found 14 species of the family Epsilonematidae and 5 species of Draconematidae, among which 10 new species and 2 new genera. The presence of an exceptional abundance of Epsilonematidae and Draconematidae was already described on the plateau of the Great Meteor Seamount [58], [59] and in deep living corals, coral degradation zones and coarse sediments [60]–[64]. Both families are associated with sediments that infills corals and sponge textures, slide over and attach to different types of substratum and are well adapted to feed on biofilms ([63] and literature therein). Such strategies are likely to be the keys to success for coping with the high turbulence regime and transient environmental conditions like the ones observed over several seamount summits.

The high dissimilarities indicate that the Condor Seamount contributes crucially to nematode β diversity. In addition, we observed large dissimilarity in terms of species composition when comparing the seamount and the external sediments (range between 77 and 96%, Fig. 5). Nematodes dissimilarity peaked between the summit and the far-field which shared only 4% of species. Although our study does not support the hypothesis that seamounts are hotspots of higher species richness when compared to the surrounding deep-sea sediments [2], it provides evidence to the hypothesis that seamounts may maintain high total biodiversity through heightened beta diversity, reflecting the turnover of faunas with depth and substrate type across the seamount [1]. Differences in sediment grain size characteristics were important in structuring nematode species composition in the Condor Seamount (42.5%, Table 6). The gravelly bioclastic sand rich in shell fragments present on the summit of the Condor Seamount [10] by providing microhabitats and niches may allow the coexistence of different species [58]. Microhabitat heterogeneity as described by sediment texture also explained differences (57.2%) in the Index of Trophic Diversity of nematodes (Table 6). Interestingly on the Condor summit, predators were dominant in the nematode assemblages due to the exclusive presence of two big nematodes (*Adoncholaimus sp.* and *Mesacanthoides sp.*), while selective deposit feeders and non selective deposit feeders characterized flanks and bases, respectively (Fig. 6). Deep-sea nematodes are mainly dominated by deposit feeders, especially the group 1A with small buccal cavities which feeds selectively on bacteria and other detrital particles [23]. However Danovaro et al. and Gambi et al. [65], [66] reported high numbers of nematode predators in the oligotrophic deep Eastern Mediterranean Sea and suggest that they may play an important role in the relationship between biodiversity and ecosystem functioning in deep sea ecosystems. Several studies showed that the inclusion of information regarding their nematode traits (e.g. the Maturity Index and the Index of Trophic Diversity) coupled with the taxonomic diversity, can provide critical information on the distribution patterns of the communities, the functioning of ecosystems and they are highly recommended in determining the environmental quality status of an ecosystem both in coastal, deep and extreme environments [67]–[72]. In our study, also the Maturity Index showed clear cut differences across the seamount; a nematode community characterized by the dominance of k-strategists in the Condor summit and the dominance of r-strategists at bases. The presence of nematodes with opportunistic life strategies is reported for coastal and deep sea nematode communities that are exposed to disturbance events [73] or to organic enriched environments [74]. The nematode maturity index on the Condor was more related to sediment trophic condition (expressed in term of phytopigments, labile compounds of the sediment organic matter). Our study showed that functional diversity of nematodes in seamounts strongly depends on environmental conditions link to the local setting and seamount structure.

## Conclusions

Habitats vary greatly over seamounts. The analysis of nematodes in soft sediment environments of the Condor Seamount distinct physiographic sites of the Condor Seamount allowed answering to the following questions:

- *Are there differences in nematode community comparing distinct physiographic sites of the Condor Seamount*?

The Condor Seamount exhibits high level of turnover comparing habitats from its base to the summit comparable to what was observed on other seamounts for megafauna [17], [75]. The unexpected presence of rich benthic assemblages at the bases of the seamounts together with the occurrence of exclusive and specialized communities in seamounts summit pose serious interrogations about the effect of present and future bottom threats such as trawling, and in particular the growing interest for the extraction of mineral resources as the polymetallic massive sulphide deposits form [19], [20], [76]–[78].

- *Is the Condor summit an area of higher nematode standing stock and a hotspot of diversity compared to other seamount habitats such as flanks and bases*?

The Condor summit harboured a completely different nematode community when compared to the other seamount physiographic sites, with a high number of exclusive species and important differences from a functional point of view. However, highest level of nematode standing stock were recorded in Condor bases, mainly due to oceanographic conditions, associated sediment mixing and high quality food resources available. It is evident that not only the summit can support a rich nematode community in terms of standing stocks and diversity and this finding should be considered in future studies on seamounts.

We conclude that only a better knowledge of the whole seamounts architecture (which should include both hard and soft substrata and different physiographic sites) and the patterns that shape their communities will help in forecasting the full extent of these impacts.
